# HYSYS: have you swapped your samples?

**DOI:** 10.1093/bioinformatics/btw685

**Published:** 2016-11-25

**Authors:** Jan Schröder, Vincent Corbin, Anthony T Papenfuss

**Affiliations:** 1 Bioinformatics Division, The Walter & Eliza Hall Institute, Parkville, Australia; 2 Computing and Information Systems, The University of Melbourne, Melbourne, Australia; 3 Department of Medical Biology, The University of Melbourne, Melbourne, Australia; 4 Peter MacCallum Cancer Centre, Melbourne, Australia; 5 Sir Peter MacCallum Department of Oncology, University of Melbourne, Melbourne, Australia

## Abstract

**Motivation:**

The application of a genomics assay to samples from a cohort is a frequently applied experimental design in cancer genomics studies. The collection and analysis of cancer sequencing data in the clinical setting is an elaborate process that may involve consenting patients, obtaining possibly-multiple DNA samples, sequencing and analysis. Many of these steps are manual. At any stage mistakes can occur that cause a DNA sample to be labelled incorrectly. However, there is a paucity of methods in the literature to identify such swaps specifically in cancer studies.

**Results:**

Here, we introduce a simple method, HYSYS, to estimate the relatedness of samples and test for sample swaps and contamination. The test uses the concordance of homozygous SNPs between samples. The method is motivated by the observation that homozygous germline population variants rarely change in the disease and are not affected by loss of heterozygosity. Our tools include visualization and a testing framework to flag possible sample swaps. We demonstrate the utility of this approach on a small cohort.

**Availability and Implementation:**

http://github.com/PapenfussLab/HaveYouSwappedYourSamples

**Supplementary information:**

Supplementary data are available at *Bioinformatics* online.

## 1 Introduction

Large cohorts of matched somatic and germline samples are used in many cancer genomics studies and there is increasing interest in cancer evolution leading to the use of multiple somatic samples. From the conception of a study, through DNA sample collection, library preparation and sequencing many manual steps are taken before the data is generated and analyzed by bioinformaticians. The manual nature of the wet lab is a source of error that is difficult to eliminate. Sample swaps, loss and contamination can occur, even in the best laboratories (Lohr *et al.*, 2015). To deal with these risks, methods are required to test for sample contamination (e.g. ContEst (Cibulskis *et al.*, 2011)) and swaps.

Here, we propose a simple strategy to establish the relatedness of possibly-multiple somatic samples with a matched germline sample in a cohort. Our method relies on data produced by SNV calling or SNP arrays. The method offers several advantages over existing methods, such as PLINK (Purcell *et al.*, 2007) or Prest (McPeek and Sun, 2000): (i) dealing with allele-specific copy number changes and loss of heterozygosity, (ii) visualising relationships between samples, (iii) automatic modeling and flagging of unusual relationships and (iv) ease of use.

## 2 Methods

There are various tests that can be employed to establish whether two samples are genetically related to each other, for example, identity-by-descent (IBD) methods (Purcell *et al.*, 2007), relatedness estimation (McPeek and Sun, 2000) and forensic testing methods. Here, we refer to the more specific case relevant in cancer cohort studies, where we need to assess whether two possibly mutated samples are from the same patient (such as primary, metastasis and germline, multiple samples, or multi-regional samples) rather than the degree of similarity or matching. However, in the context of cancer, IBD methods can suffer from the change in allele frequencies caused by copy number changes, which occur on a large scale in many cancers (Shlien and Malkin, 2009). Therefore, our method is designed to be robust to such rearrangements by focusing on homozygous germ line mutations. The motivation is as follows: a cancer descends from germ line cells and therefore shares all variants in the early stages. In the case of a *homozygous* germline variant, it is unlikely that the cancer would carry any other genotype—amplifying a chromosome or parts thereof does not change the frequency if there is only one allele to begin with; neither does losing a copy (except for the loss of both copies of a chromosome). Structural variants, such as inversions, translocations or other amplifications do not disturb such variants either (only indirectly by disrupting mappability around variants, but those are local phenomena).

Our proposed method is simply taking the concordance of homozygous germ line variants in the sample as a metric of relatedness. This metric allows sample relationships to be visualized using simple heat map plots to quickly verify that all samples that should be paired in a cohort are indeed paired. Further, it allows a simple statistical model to be created on the metric data as it follows a fairly tractable bimodal distribution. Such a model can identify any significant outliers to allow assessment of potential problems of contamination or unpaired samples within the cohort.

More specifically, our cohort analysis method offers three modules:

*Concordance calculation:* The first module takes samples and controls (or only samples) as input and calculates the concordance metric as outlined above. The results are written to an output file for further analysis.

**Fig. 1.**
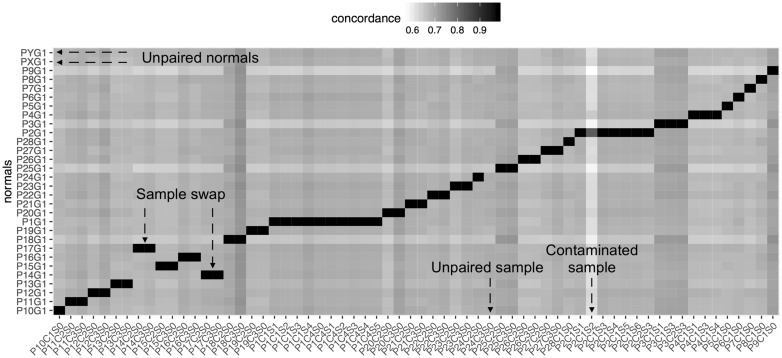
Heat map plot of concordance values in a cohort of 70 gastric cancers and matched normals. Annotations in red highlight the specific issues with the cohort that can be visually identified

*Heat map plotting:* The second module takes the output from the first to generate a graphical representation of the results. An example of such a plot can be seen in Figure 1.

*Statistical modeling and flagging:* The final module creates a mixed model of Gaussian distributions from the concordance data and then establishes for every pair of sample and control (or sample, sample) whether they are (i) unrelated, (ii) related, or (iii) neither. The latter case may occur if a sample has been contaminated with unrelated DNA causing a change of genotype in some of the homozygous variants (see Supplementary Fig. S1). Contamination can be further estimated with tools such as ContEst by (Cibulskis *et al.*, 2011).

## 3 Results

The example heat map in Figure 1 shows the graphical representation of the concordance values for a cohort of gastric cancer samples. A separation of related and unrelated samples is obvious due to the colouring. A sample/normal swap is also easily spotted due to the sort order of samples and normals and a shift away from the diagonal (bottom left). Furthermore, there are two normal and one sample that are not paired up (identifiable by dark rows/column). Finally, there is a distinctly dark column in patient 2 (P2C1S2).

The statistical modeling of the data through a mixture of Gaussian functions allows the automation of data analysis. The model components are described in more detail in the supplement and plotted in Figure S1. This module of our method automatically identifies samples P2C1S2 and P24C3S0 as being non-paired within the data. It also flags the concordance values of P2C1S2 and its expected pairings as abnormal under the model (the sample has since been confirmed to be contaminated by another patient’s DNA). In contrast, when applying the same modeling technique to the results obtained by PLINK’s IBD testing, only the unpaired sample P24C3S0 can be identified, but the bimodal distribution of the IBD-sharing does not allow for confident identification of values that lie distinctly between the two peaks.

## 4 Discussion

We developed a method that allows for a quick automatic and visual sanity check of relationships within cancer cohorts. While similar results could be achieved using IBD methods (albeit with less sensitivity to uncommon relationship values), our approach allows for a statistical model that identifies outlying values more reliably, and offers higher ease of use.

## Supplementary Material

Supplementary Data
