# Supplementary material for: HYSYS: have you swapped your samples?
Source: Bioinformatics. 2016 Nov 25;33(4):596–8. doi: 10.1093/bioinformatics/btw685 (PMC5408803; doi:10.1093/bioinformatics/btw685)
Supplement: Supplementary Data [file btw685_supp.docx]

# HYSYS: Have You Swapped Your Samples?

Jan Schröder, Vincent Corbin and Anthony T. Papenfuss^*^

# Supplementary Material

## Data

The data used to demonstrate HYSYS consist of 70 samples of gastric cancer DNA from 30 patients. Samples underwent whole exome sequencing and variants were called using Mutect for the somatic samples and VarScan2 for germline samples, predicting around 150,000 variants each. HYSYS can operate with other types of sequencing or genotyping protocols, as it only depends on homozygous variant calls. It requires 10s to 100s of homozygous SNPs to measure their relationship effectively.

## Statistical Modelling

Analysing different cohorts of variant calls, we have observed very distinct bimodal distributions for the concordance measure. Further, the two peaks of the two distributions look reasonably close to normally distributed (see for example Figure S1). We therefore integrated a simple Gaussian mixture model into our analysis of the concordance data. There are numerous software packages released that offer such functionality and we chose the Python module *GMM* (Gaussian Mixture Model) within the popular *scikit* package.

The GMM is set to fit two components and reports the means and variances for each as well as the weights (the proportion of values falling under each distribution). Figure S1 shows the density plot for the raw data of concordance measures as well as the two components of the fitted mixture model in red (unrelated samples) and blue (related samples). In this example the peaks of the model are clearly separated and are therefore suitable for automatic detection of related and unrelated samples.

We use the model to score each data point, which assigns a probability that the data was generated by either model component. The script runs through all sample/normal concordance values and reports any related pairs as well as concordance values that are rejected under the model (data points that fit neither model component (with a cutoff of 0.05). The latter allows the user to identify odd data points such as the ones generated by contamination in our example (the small blip at about 0.82 concordance in Figure S1). Note, the 0.05 cutoff is not corrected for multiple testing. From our experience the model tends to have quite wide peaks anyway, so a further correction of the tail would likely lead to the distributions overlapping.


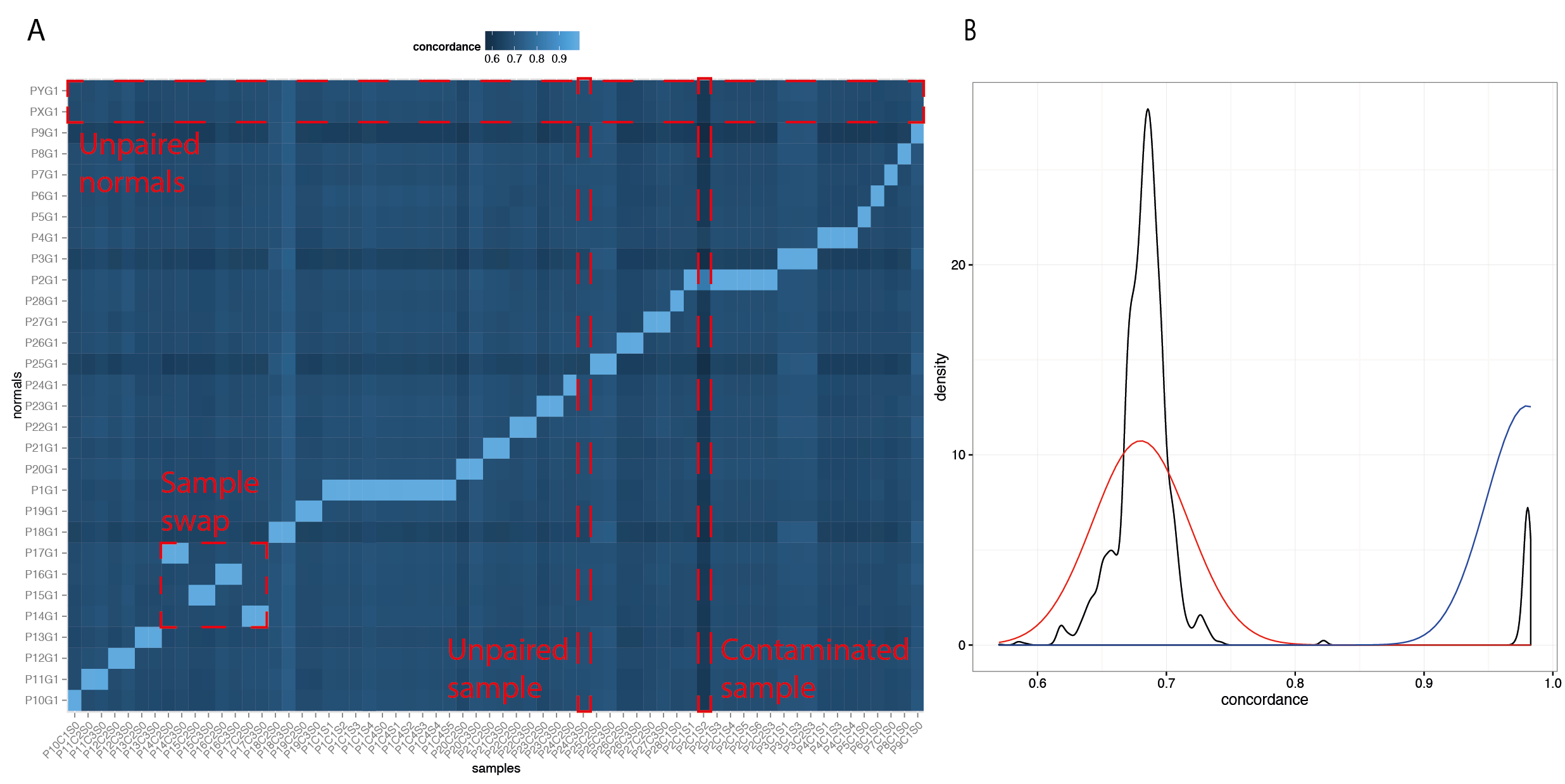


Figure S1: Density function of concordance values (in black) and the Gaussian mixture model for unrelated samples (red) and related samples (blue).
